# Expression of tSTAT3, pSTAT3^727^, and pSTAT3 ^705^ in the epithelial cells of hormone‐naïve prostate cancer

**DOI:** 10.1002/pros.23787

**Published:** 2019-03-24

**Authors:** Agnieszka Krzyzanowska, Nicholas Don‐Doncow, Felicia Elena Marginean, Alexander Gaber, R. William Watson, Rebecka Hellsten, Anders Bjartell

**Affiliations:** ^1^ Department of Translational Medicine, Division of Urological Cancers Lund University Malmö Sweden; ^2^ Department of Clinical Sciences, Division of Pathology Lund University Lund Sweden; ^3^ UCD School of Medicine, Conway Institute of Biomolecular and Biomedical Research University College Dublin Belfield Dublin Ireland; ^4^ Department of Urology Malmö University Hospital Malmö Sweden

**Keywords:** biomarker, immunohistochemistry, prostate cancer, signal transducer and activator of transcription 3, tissue microarray

## Abstract

**Background:**

The signal transducer and activator of transcription 3 (STAT3) pathway is observed to be constitutively activated in several malignancies including prostate cancer (PCa). In the present study, we investigated the expression of total STAT3 (tSTAT3) and two forms of activated phosphorylated STAT3 (pSTAT3^727^ and pSTAT3^705^) in tissue microarrays (TMA) of two cohorts of localized hormone‐naïve PCa patients and analyzed associations between the expression and disease outcome.

**Methods:**

The expression of tSTAT3, pSTAT3^727^, and pSTAT3^705^ was scored in the nuclei and cytoplasm of prostatic gland epithelial cells in two TMAs of paraffin‐embedded prostatic tissue. The TMAs consisted of tissue originated from hormone‐naïve radical prostatectomy patients from two different sites: Malmö, Sweden (*n* = 300) and Dublin, Ireland (*n* = 99).

**Results:**

The nuclear expression levels of tSTAT3, pSTAT3^727^, and pSTAT3^705^ in the epithelial cells of benign glands were significantly higher than in the cancerous glands. Cytoplasmic tSTAT3 levels were also higher in benign glands. Patients with low pSTAT3^727^ and pSTAT3^705^ levels in the cancerous glands showed reduced times to biochemical recurrence, compared with those with higher levels. No significant trends in nuclear nor in cytoplasmic tSTAT3 were observed in relation to biochemical recurrence in the Malmö cohort. Higher cytoplasmic tSTAT3 was associated with reduced time to biochemical recurrence in the Dublin cohort. Adding the tSTAT3 and pSTAT3 expression data to Gleason score or pathological T stage did not improve their prognostic values.

**Conclusions:**

Low pSTAT3^727^ and pSTAT3^705^ expression in epithelial cells of cancerous prostatic glands in hormone‐naïve PCa was associated with faster disease progression. However, pSTAT3 and tSTAT3 expression did not improve the prognostic value of Gleason score or pathological T stage and may not be a good biomarker in the early hormone naïve stages of PCa.

## 1 INTRODUCTION

Prostate cancer (PCa) is the most commonly diagnosed cancer in men and is the second leading cause of death from cancer in men.^1^ At diagnosis, PCa is usually confined to the prostate and less than one‐third of patients will actually die from the PCa.^1^ An increasing number of men with localized PCa are being followed by active surveillance, or being offered curative treatment with radical prostatectomy or radiation therapy at disease progression.^2^ To optimize active surveillance, there is a need to identify, at an early stage, those patients who are at a lower risk of developing a more advanced disease and would not benefit from invasive treatments. New prognostic biomarkers are therefore necessary.

The transcription factor signal transducer and activator of transcription 3 (STAT3) is an important oncogenic‐associated protein and found to be constitutively activated by phosphorylation in several malignancies including PCa.^3^, ^4^, ^5^, ^6^, ^7^, ^8^ STAT3 is activated by a number of inflammatory cytokines such as interleukin 6 (IL‐6), IL‐10, IL‐11, and IL‐21. Other factors secreted within the tumor, such as vascular endothelial growth factor, epidermal growth factor, and platelet‐derived growth factor may also activate STAT3.^9^, ^10^ High serum levels of IL‐6 in PCa patients have been implicated in lower survival rates.^11^ Activation of STAT3 by phosphorylation on the 705 tyrosine or the 727 serine has been observed to be involved in cancer progression and a more aggressive phenotype of PCa.^12^ However, STAT3 may in certain contexts act as a tumor suppressor 10 and new evidence is emerging showing antioncogenic roles of the STAT3‐IL‐6 pathway in PCa.^13^

The studies investigating the expression patterns of total STAT3 (tSTAT3) and phosphorylated STAT3 (pSTAT3^727^ and pSTAT3^705^) in various stages of PCa are limited. We have previously observed high expression levels of pSTAT3^705^ in PCa metastases from castration‐resistant PCa patients^14^ and in the present study we aim to investigate the expression of tSTAT3, pSTAT3^727^, and pSTAT3^705^ in localized hormone naïve PCa to evaluate their expression in early stage cancer and their value as prognostic biomarkers.

## 2 MATERIALS AND METHODS

### 2.1 Patient cohorts—Hormone naïve patients with localized PCa

#### 2.1.1 Malmö cohort

A tissue microarray (TMA) was constructed using a previously described protocol^15^ from a population‐based cohort of 341 PCa patients who underwent open radical prostatectomy between 1998 and 2006 at the Department of Urology, Skåne University Hospital, Malmö, Sweden. Two malignant and two distant benign cores from each patient were mounted in paraffin blocks. A senior National Board certified pathologist (FM) scored each individual core for Gleason score using hematoxylin & eosin stained tissue sections. The clinical and pathological characteristics of the PCa patients were obtained from reading the patient charts and are shown in Table 1. The mean follow‐up time was 130 months (range, 13‐220). Since there was a very small percentage of PCa‐related deaths, biochemical recurrence (BCR) was used as an endpoint for outcome measurement, defined by a rise in the blood prostate‐specific antigen (PSA) level to at least 0.2 ng/mL with a subsequent confirmatory value. Missing tissue cores, staining artifacts and patients receiving any hormonal or chemotherapy treatment prior to before surgery were removed from analysis—final numbers are provided in the figures. The study has been approved by the Local Ethic's committee at Lund University no. 494/2005.

**Table 1:** Patient characteristics in two different cohorts

	Malmö	Dublin
*Age at time of surgery, y*		
Mean (median) (%)	62.7 (63)	60.5 (61)
<50	5 (1.7)	8 (8.1)
50‐59	72 (24.0)	35 (35.4)
60‐69	197 (65.7)	51 (51.5)
>70	26 (8.7)	5 (5.1)
Clinical stage, (%)		
cT1c	180 (60)	
cT2	111 (37)	
cT3	4 (1.3)	
unknown	5 (1.7)	
Prostatectomy Gleason score (ISUP grade) (%)		
Grade 1 (≤6)	135 (45.0)	26 (26.3)
Grade 2 (3 + 4)	100 (33.3)	30 (30.3)
Grade 3 (4 + 3)	47 (15.7)	17 (17.2)
Grade 4 and 5 (≥8)	15 (5.0)	26 (26.3)
Unknown	2 (0.7)	
Pathological stage (%)		
pT2	156 (52.0)	51 (51.5)
pT3	136 (45.3)	48 (48.5)
pT4	1 (0.3)	
Unknown	7 (2.3)	
*Positive surgical margins*	146 (49)	46 (46.5)
PSA at diagnosis, ng/mL		
Mean (median)	8.8 (7.1)	8.6 (8)
Range	2.6‐35.1	1‐18.8
Follow‐up, mo		
Mean (median)	128.9 (129.5)	51.9 (53.0)
Range	13‐220	2‐116
Overall no. of biochemical recurrences (%)	88 (29)	48 (49)

#### 2.1.2 Dublin cohort

The TMA was constructed in a similar way to that described above from 99 PCa patients who underwent open radical prostatectomy between 2003 and 2010 at three referral hospitals in Dublin, collected as part of the Prostate Cancer Research Consortium bioresource^16^ following informed written consent. Up to nine cores were available from each patient: three from a benign region, three from a lower grade (Gleason grade 3) and three from a higher grade (Gleason grades 4 or 5) region. The clinical and pathological characteristics of the patients are summarized in Table 1. The mean follow‐up time was 51.9 months (range, 2‐116). BCR was used as an endpoint for outcome measurement, defined by a rise in the blood PSA level to at least 0.4 ng/mL with a subsequent confirmatory value. The final number of patients used in the present study was 99 and the number of patients with BCR (*n* = 48) was matched with patients with no BCR (*n* = 51). The study has been approved by the Local Ethic's committee reference number 1/378/660.

### 2.2 Immunohistochemistry

**Figure 1:**
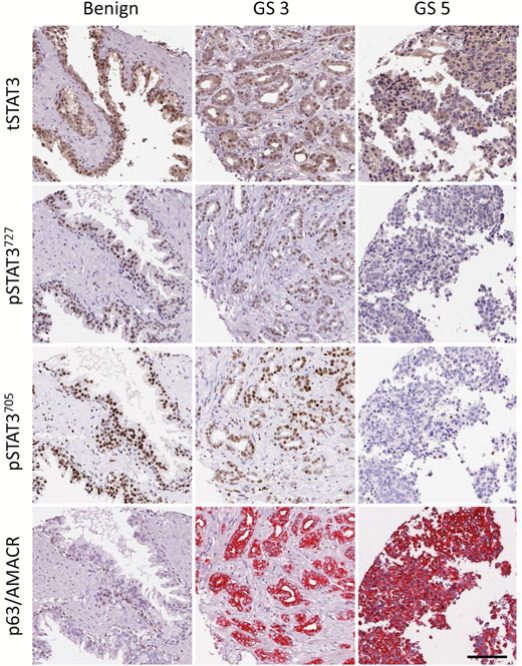
Examples of tSTAT3, pSTAT3^727^, pSTAT3^705^, and p63/AMACR immunostainings in consecutive sections of benign cores and cores with Gleason pattern 3 (GS3) and 5 (GS5). Scale bar = 100 µm. AMACR, α‐methyl acyl‐CoA racemase; pSTAT3, phosphorylated STAT3; STAT3, signal transducer and activator of transcription 3; tSTAT3, total STAT3 [Color figure can be viewed at wileyonlinelibrary.com]

Tissue sections were cut in 4 µM sections from paraffin blocks and mounted onto slides. Sections underwent preprocessing where they were deparaffinized with xylene and ethanol followed by rehydration and antigen retrieval. Antigen retrieval of the tissue sections was performed using a PT‐Link module (DAKO, Glostrup, Denmark) at 95°C to 99°C for 20 minutes (pH 9.0). The sections were then stained in a DAKO Autostainer‐plus using the EnVision FLEX including Peroxidase‐Blocking Reagent (DAKO). Consecutive sections of the TMAs were immunostained for p63 (M7001, 1:50; DAKO) + AMACR (α‐methyl acyl‐CoA racemase [M3616, 1:100; DAKO], tSTAT3 [8019, 1:50 Santa Cruz Biotechnology, Dallas, TX], pSTAT3 phosphorylated at serine 727 [9134, 1:100; Cell Signaling Technology, Danvers, MA], and pSTAT3 phosphorylated at tyrosine 705 [76315, 1:100; Abcam, Cambridge, UK]). Controls were performed to verify the antibody specificity (Figure S1). The p63/AMACR double staining allowed specific identification of benign versus tumor areas by the visualization of the nuclear p63‐positive basal cells and cytoplasmic AMACR‐positive tumor cells. Examples of the four different immunostainings are shown in Figure 1.

### 2.3 Scoring procedure

**Figure 2:**
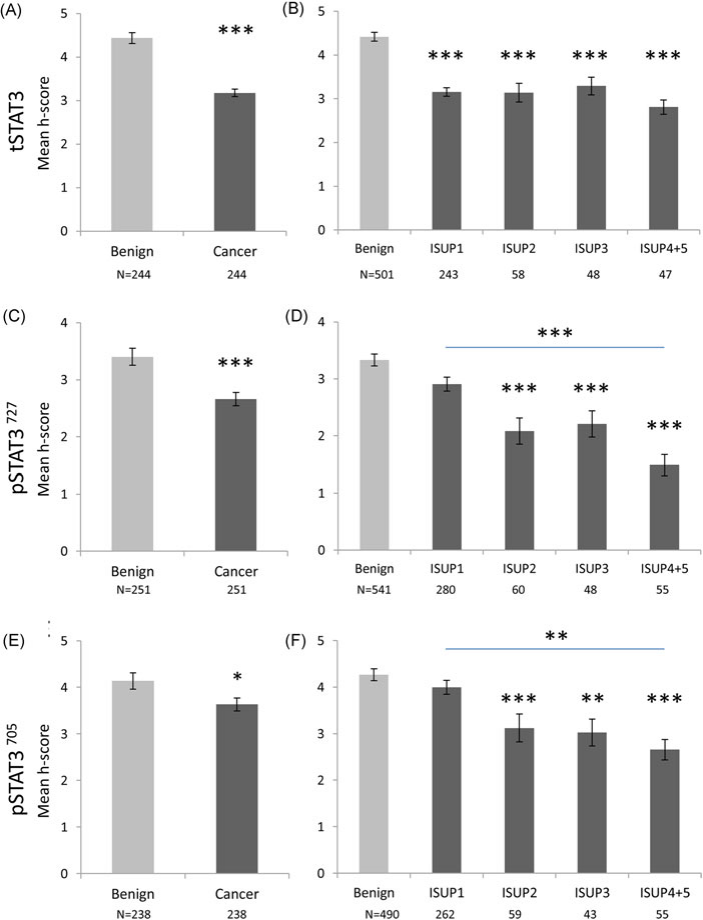
Nuclear expression of tSTAT3 (A,B), pSTAT3^727^ (C,D), and pSTAT3^705^ (E,F) in the benign and malignant prostatic epithelium, in the Malmö cohort. A,C, and E, average *H* score for all benign cores vs all cancer cores. Paired analysis (the Wilcoxon signed‐rank test) showed decreased *H* score for all markers in the cancer cores. Numbers of patients are shown underneath the graphs. B,D, and F, stratification of individual cores by ISUP 2014 Gleason grade classification. A progressive decrease in mean *H* score in higher Gleason grades can be observed for all markers (unpaired cores, one‐way ANOVA on Ranks). Number of cores shown below graph. One or two cores were available in each category from each patient. Data are represented as mean ± SEM. All significances indicated are between benign and other group, unless otherwise stated, *P < 0.05, **P < 0.01,***P < 0.001. ANOVA, analysis of variance; pSTAT3, phosphorylated STAT3; STAT3, signal transducer and activator of transcription 3; tSTAT3, total STAT3 [Color figure can be viewed at wileyonlinelibrary.com]

**Figure 3:**
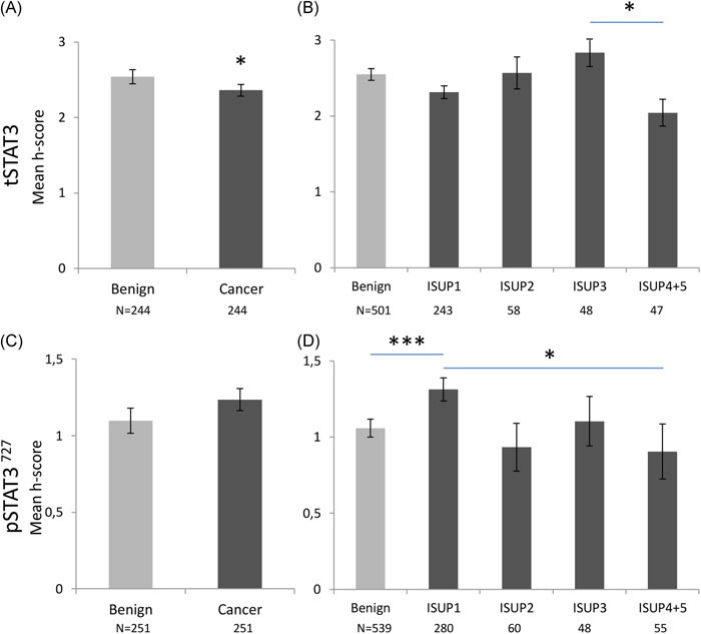
Cytoplasmic expression of tSTAT3 and pSTAT3^727^ in the prostatic epithelium, in the Malmö cohort. No cytoplasmic pSTAT3^705^ staining was observed. A,C, Average *H* score for all benign cores vs all cancer cores. Paired analysis (the Wilcoxon signed‐rank test) showed differences between average tSTAT3 *H* score in benign and cancer cores. Numbers of patients are shown underneath the graphs. B,D, Stratification of individual cores by ISUP 2014 Gleason grade classification. An elevated expression of pSTAT3 727 was observed in grade 1 (GS < 7) cores (unpaired cores, one‐way ANOVA on ranks). Number of cores shown below graph. One or two cores were available in each category from each patient. Data are represented as mean ± SEM. All significances indicated are between benign and other group, unless otherwise stated, *P < 0.05, **P < 0.01,***P < 0.001. ANOVA, analysis of variance; pSTAT3, phosphorylated STAT3; STAT3, signal transducer and activator of transcription 3; tSTAT3, total STAT3 [Color figure can be viewed at wileyonlinelibrary.com]

**Figure 4:**
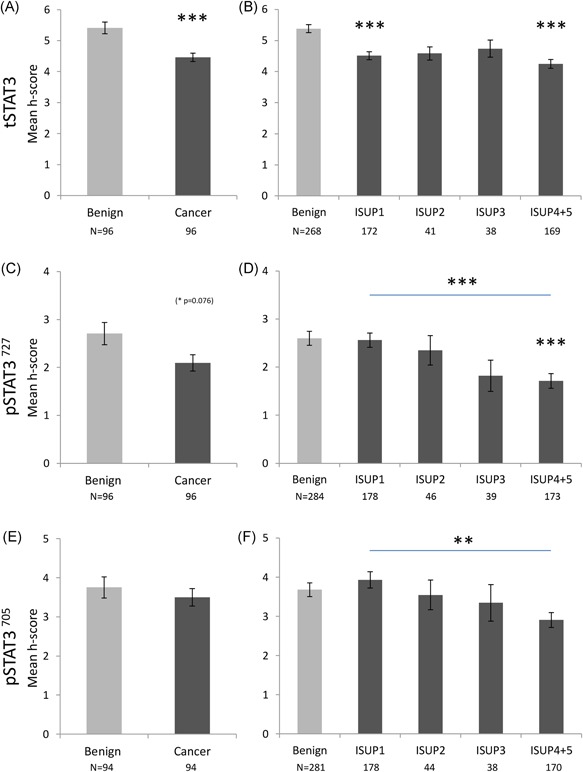
Nuclear expression of tSTAT3 (A,B), pSTAT3^727^ (C,D), and pSTAT3^705^ (E,F) in the benign and malignant prostatic epithelium, in the Dublin cohort. A,C, and E, Average *H* score for all benign cores vs all cancer cores. Paired analysis (the Wilcoxon signed‐rank test) showed decreased *H* score for tSTAT3 in the cancer cores (*P* < 0.001). Numbers of patients are shown underneath the graphs. B,D, and F, Stratification of individual cores by ISUP 2014 Gleason grade classification. A progressive decrease in mean *H* score in higher Gleason grades can be observed for all markers (unpaired cores, one‐way ANOVA on ranks). Number of cores shown below graph. One to three cores were available in each category from each patient. Data are represented as mean ± SEM. All significances indicated are between benign and other group, unless otherwise stated, **P < 0.01,***P < 0.001. ANOVA, analysis of variance; pSTAT3, phosphorylated STAT3; STAT3, signal transducer and activator of transcription 3; tSTAT3, total STAT3 [Color figure can be viewed at wileyonlinelibrary.com]

**Figure 5:**
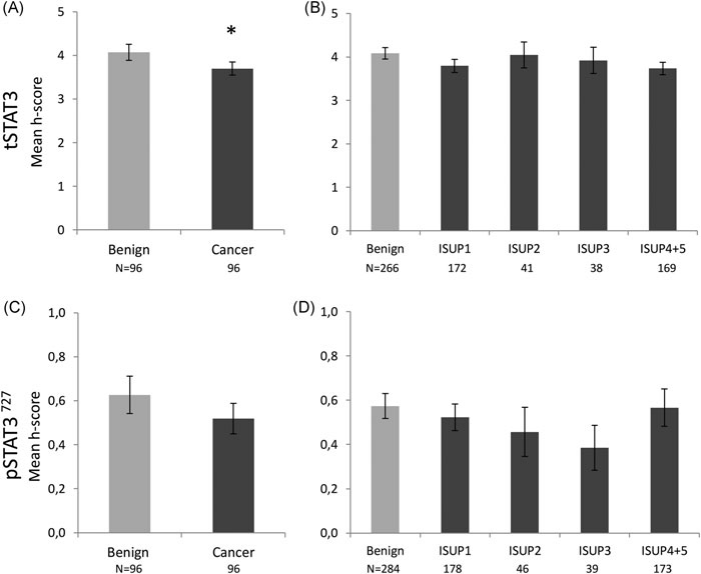
Cytoplasmic expression of tSTAT3 and pSTAT3^727^ in the prostatic epithelium, in the Dublin cohort. No cytoplasmic pSTAT3^705^ staining was observed. A,C, Average *H* score for all benign cores vs all cancer cores. Paired analysis (the Wilcoxon signed‐rank test) showed a significant difference on average tSTAT3 *H* score between benign and cancer cores (*P* < 0.05). Numbers of patients are shown underneath the graphs. B,D, Stratification of individual cores by ISUP 2014 Gleason grade classification. No significant differences were observed between the different Gleason grades (unpaired cores, one‐way ANOVA on ranks). Number of cores shown below graph. One to three cores were available in each category from each patient. Data are represented as mean ± SEM. ANOVA, analysis of variance; pSTAT3, phosphorylated STAT3; STAT3, signal transducer and activator of transcription 3; tSTAT3, total STAT3

Slides were scanned using an Aperio CS2 slide scanner and images were viewed on the Aperio ImageScope Software (Leica Biosystems, Wetzlar, Germany). The intensity of the nuclear and cytoplasmic staining in the glandular epithelial cells was manually recorded as a score between 0 and 3 (zero, low, moderate, and high; Figure S2) and the percent of nuclei stained was also recorded (<10% = 1, 11‐75% = 2, >75% = 3). The intensity score and the fraction of positively stained cells were multiplied to give a final score (*H* score, 0‐9, adapted from Detre et al^17^) that was then used as a representation of expression level in each given patient. A consensus between the scorer (AK) and an experienced pathologist (FM) was reached before the scoring. The results were based on the average score of two benign and two cancer cores from each patient in the Malmö cohort. For the Dublin cohort, the average of three benign cores and up to six cancer cores per patient was used. In the case of missing cores, the score of one core was used. For Figures 2B, 2D and 2F; 3B and 3D; 4B, 4D and 4F; 5B and 5D, the analysis was done on a per‐core basis.

### 2.4 Statistical analysis

**Figure 6:**
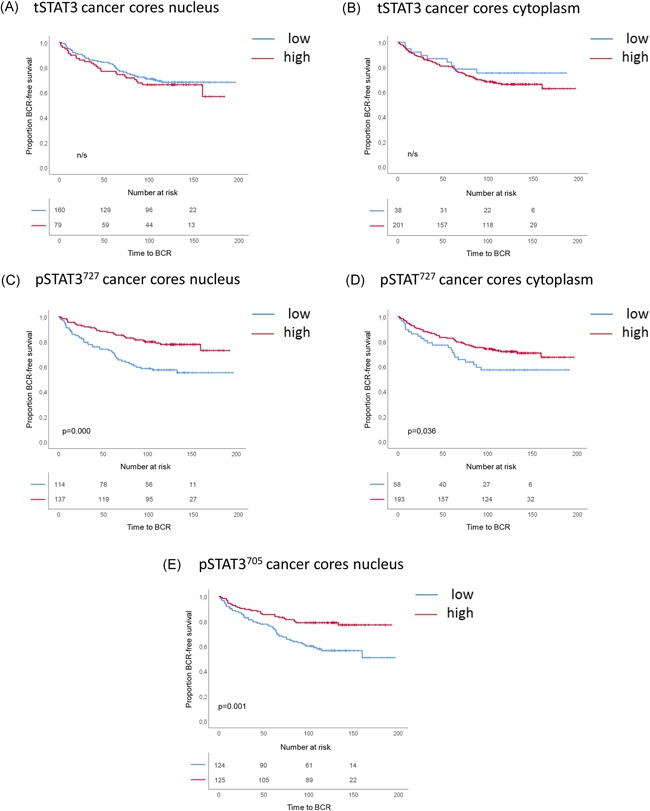
Kaplan‐Meier curves of BCR‐free survival in the Malmö cohort for expression of: (A) nuclear tSTAT3, (B) cytoplasmic tSTAT3, (C) nuclear pSTAT3^727^, (D) cytoplasmic pSTAT3^727^, and (E) nuclear pSTAT3^705^. All graphs are based on expression in cancer cores. Cutoff between high and low *H* score was calculated using the Youden index. Log‐rank test statistic was used to determine the *P* value. BCR, biochemical recurrence; pSTAT3, phosphorylated STAT3; STAT3, signal transducer and activator of transcription 3; tSTAT3, total STAT3 [Color figure can be viewed at wileyonlinelibrary.com]

**Figure 7:**
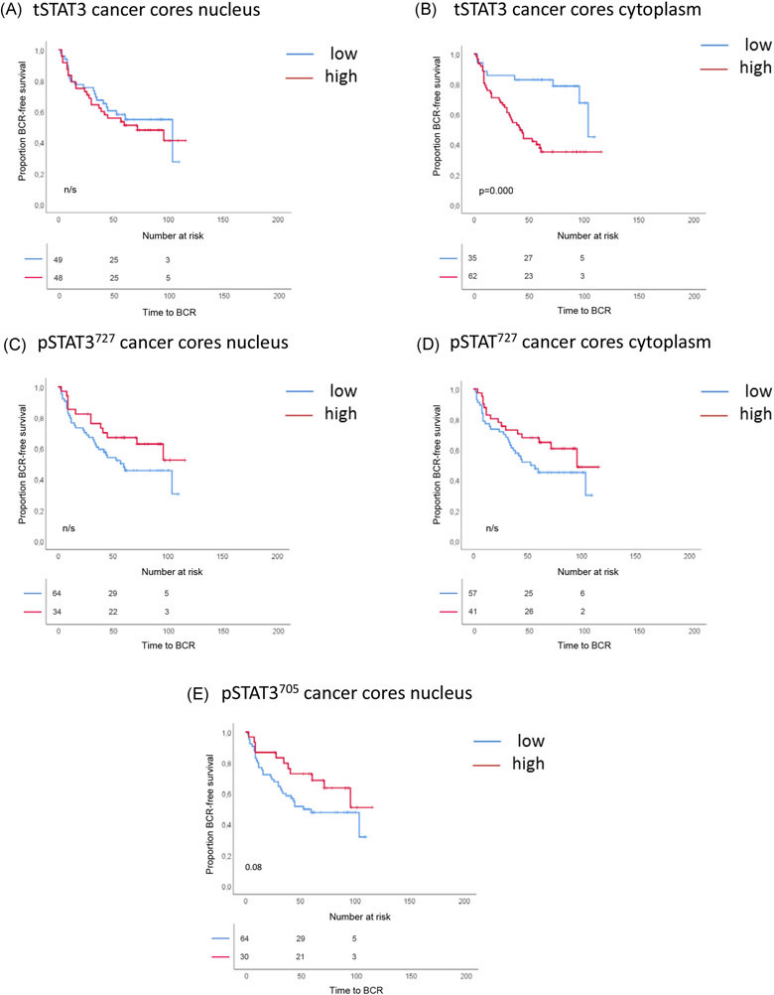
Kaplan‐Meier curves of BCR‐free survival in the Dublin cohort for expression of: (A) nuclear tSTAT3, (B) cytoplasmic tSTAT3, (C) nuclear pSTAT3^727^, (D) cytoplasmic pSTAT3^727^, and (E) nuclear pSTAT3^705^. All graphs are based on expression in cancer cores. Cutoff between high and low *H* score was calculated using the Youden index. Log‐rank test statistic was used to determine the *P* value. pSTAT3, phosphorylated STAT3; STAT3, signal transducer and activator of transcription 3; tSTAT3, total STAT3 [Color figure can be viewed at wileyonlinelibrary.com]

Statistical analysis was performed using SPSS (IBM, Armonk, NY) and R (The R Foundation, https://www.r‐project.org/). The mean intensity scores of the benign and cancer cores were compared using the Wilcoxon signed‐rank test for paired samples or analysis of variance (ANOVA) on ranks for comparing groups. Spearman correlation coefficient (*r*_s_) was used for calculating correlations. Kaplan‐Meier curves were performed for tSTAT3, pSTAT3^727^, and pSTAT3^705^. The best cutoff was calculated using the Youden's J statistic for each category and this value was used to dichotomize the data into “low” and “high” as presented in Figures 6 and 7. The cutoffs for the Kaplan‐Meier curves for pathological Gleason score (pGS) were: <7 (ISUP1), 3 + 4 (ISUP2), 4 + 3 (ISUP3) and >7 (ISUP 4 and 5) and for pathological T stage (pT): pT2 and pT3. Log‐rank test statistic was used to determine the *P* value. Multivariable Cox regression was used to determine predictive values of the different markers.

## 3 RESULTS

### 3.1 Immunostaining

#### 3.1.1 Malmö cohort

Nuclear expression of tSTAT3, pSTAT3^727^, and pSTAT3^705^ in the Malmö cohort was significantly lower in the cancerous epithelium, compared with the benign epithelium (the Wilcoxon signed‐rank test, *P* < 0.001 for tSTAT3, pSTAT3^727^ and *P* < 0.05 for pSTAT3^705^, Figure 2A, 2C, and 2E). Stratification of the expression according to the Gleason score of the individual cores showed that the tSTAT3 *H* score was lower in all cancer cores, compared with benign cores (Figure 2B) and for pSTAT3^727^ and pSTAT3^705^, the *H* score progressively decreased with increasing Gleason score (Figure 2D and 2F, ANOVA on ranks).

Cytoplasmic expression was observed only for tSTAT3 and pSTAT3^727^ and not for pSTAT3^705^ (Figure 3). tSTAT3 showed lower average cytoplasmic expression in cancer cores, compared with benign cores from the same patients (the Wilcoxon signed‐rank test, *P* < 0.05; Figure 3A). There was no significant difference in pSTAT3^727^ expression between benign and cancer (Figure 3C), but, when stratified according to Gleason score, higher expression was observed for pSTAT3^727^ in GS < 7 cores (ISUP grade 1; Figure 3D, ANOVA on ranks).

#### 3.1.2 Dublin cohort

The nuclear expression of the three markers followed a similar pattern in the Dublin cohort, in that the *H* score in the cancer cores was lower than in the benign cores (Figure 4). When comparing benign and cancer cores from the same patient, only the difference in tSTAT3 expression was significant (Figure 4A, *P* < 0.001, the Wilcoxon signed‐rank test, *n* = 96). There was a tendency for the pSTAT^727^ *H* score to be lower in the cancer cores (Figure 4C, *P* = 0.076, the Wilcoxon signed‐rank test, *n* = 96). Stratifying the cores according to their Gleason score, showed a progressive decrease in nuclear expression intensities for all three markers in the higher Gleason scores (Figure 4B, 4D, and 4F, ANOVA on ranks).

As in the Malmö cohort, only tSTAT3 and pSTAT^727^ showed cytoplasmic staining. There was a significant difference between benign and cancer cores from the same patient for cytoplasmic tSTAT3 (*P* = 0.04, the Wilcoxon signed‐rank test, *n* = 96; Figure 5A), but there were no significant differences amongst the different Gleason scores.

### 3.2 Correlations

Table 2 shows correlations (Spearman correlation, *r*_s_) between the different markers in both nucleus and cytoplasm. Moderate correlation was assumed to be above 0.5 and high above 0.7.^18^ We observed high correlations between nuclear pSTAT3^727^ and pSTAT3^705^ in both benign (*r*_s_ = 0.77) and cancer (*r*_s_ = 0.71) cores in the Malmö cohort and even higher in the Dublin cohort (*r*_s_ = 0.85 and 0.82, respectively). Nuclear tSTAT3 correlated highly with nuclear pSTAT3^727^ (Malmö: *r*_s_ = 0.81, Dublin: *r*_s_ = 0.65) and nuclear pSTAT3^705^ (Malmö: *r*_s_ = 0.67, Dublin: *r*_s_ = 0.71) in the benign cores but the correlation was much lower in the cancer cores (*r*_s_ < 0.35 in Malmö and <0.61 in Dublin). Cytoplasmic tSTAT3 correlated with cytoplasmic pSTAT3^727^ (Malmö: *r*_s_ = 0.53, Dublin: *r*_s_ = 0.56) in the benign cores but the correlation was much lower in the cancer cores (*r*_s_ < 0.24). Nuclear and cytoplasmic expression was moderately correlated in both tSTAT3 (Malmö: *r*_s_ = 0.64 benign, *r*_s_ = 0.43 cancer; Dublin: *r*_s_ = 0.57 benign, *r*_s_ = 0.38 cancer) and pSTAT3^727^ (Malmö: *r*_s_ = 0.69 benign, *r*_s_ = 0.62 cancer; Dublin: *r*_s_ = 0.74 benign, *r*_s_ = 0.65 cancer). However, the correlation of the same marker in between cancer and benign cores was very poor for all three markers, in both nuclear and cytoplasmic compartments (*r*_s_ < 0.21 in Malmö and *r*_s_ < 0.43 in Dublin cohorts).

**Table 2:** Correlations between the different markers

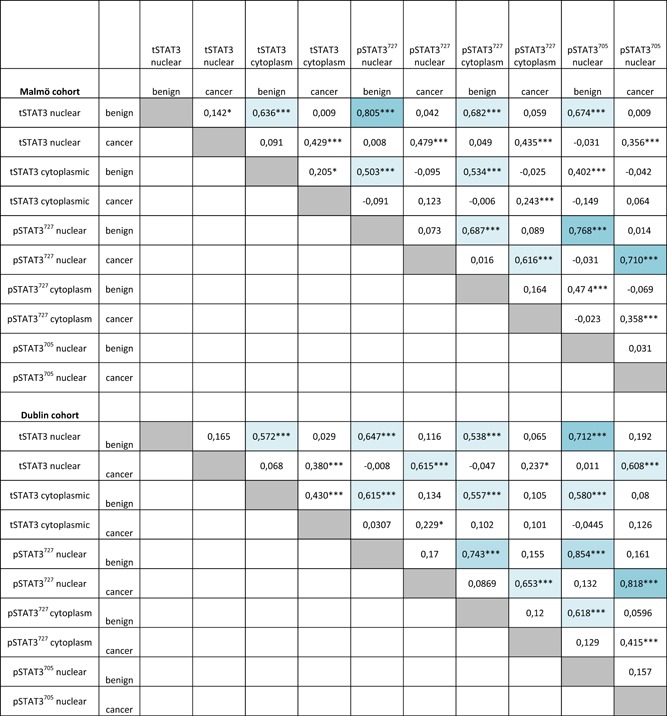

Dark blue indicates correlation coefficient of >0.7, light blue, correlation coefficient of >0.5.

*P < 0.05.

**P < 0.01.

***P < 0.001—Spearman correlation coefficient.

### 3.3 Outcome analysis

Nuclear and cytoplasmic tSTAT3 expression in the Malmö cohort was not predictive of BCR progression (Figure 6A and 6B). However, in the Dublin cohort, patients with higher cytoplasmic tSTAT3 had a shorter time to BCR (*P* < 0.001, Figure 7B).

For Malmö pSTAT3^727^, both low nuclear (Figure 6C) and low cytoplasmic (Figure 6D) expression predicted worse outcome (*P* < 0.001 and *P* < 0.05). Similar patterns were observed in the Dublin cohort, although they did not reach significance (Figure 7C and 7D).

Malmö patients with low pSTAT3^705^ nuclear expression in the cancer gland epithelial cells had a shorter time to BCR (*P* < 0.001; Figure 6E). The Dublin data followed the same trend (Figure 7E, *P* = 0.08).

No significant predictive trends were observed for any of the three markers in benign cores (data not shown).

Survival analysis based on pathological tumor (pT) stage produced Kaplan‐Meier curves which demonstrated that patients with pT2 had longer time to BCR compared with pT3 in both cohorts (Figure S3A and SC). Survival benefit was also seen in relation to pGS at radical prostatectomy in the Malmö cohort—lower pGS resulted in longer time to BCR (Figure S3B and SD).

In the Malmö cohort, pGS and pT were predictive of progression (Table 3). Nuclear pSTAT3^727^ and pSTAT3^705^ in the cancer cores were also predictive (*P* < 0.05, Table 3) but multivariable analysis of these markers did not improve the prognostic value of pGS or pT stage. In the Dublin cohort, pGS, pT and cytoplasmic tSTAT3 were predictive of progression (*P* < 0.05, Table 3). Multivariable analysis did not improve the prognostic value of pGS or pT stage.

**Table 3:** Cox univariable and multivariable analysis of BCR‐free survival according to the biomarker expression in cancer cores, Gleason score, and pathological T stage

	*N* (events)	Univariable HR(95% CI)	Multivariable HR^§^
Malmö TMA					
Biomarker (continuous)			
tSTAT3 nucleus	239 (75)	0.95 (0.80‐1.13)	1.15 (0.90‐1.49)
tSTAT3 cytoplasm	239 (75)	1.00 (0.83‐1.20)	0.97 (0.77‐1.22)
pSTAT3^727^ nucleus	251 (77)	0.83 (0.72‐0.94)**	0.90 (0.71‐1.15)
pSTAT3^727^ cytoplasm	251 (77)	0.85 (0.68‐1.06)	0.91 (0.67‐1.22)
pSTAT3^705^ nucleus	249 (78)	0.88 (0.79‐0.98)*	0.93 (0.79‐1.10)
Pathological Gleason score			
Low—ISUP 1 and 2	230 (56)	Reference	Reference
High—ISUP 3, 4, and 5	61 (31)	2.60 (1.67‐4.03)***	2.26 (1.38‐3.17)***
Pathological T stage			
Low ≤pT2	154 (24)	Reference	Reference
High ≥pT3	133 (61)	3.54 (2.20‐4.67)***	2.67 (1.57‐4.54)***
Dublin TMA			
Biomarker (continuous)			
tSTAT3 nucleus	97 (47)	1.06 (0.86‐1.31)	0.99 (0.69‐1.42)
tSTAT3 cytoplasm	97 (47)	1.28 (1.07‐1.55)**	1.24 (0.99‐1.56)
pSTAT3^727^ nucleus	98 (47)	0.87 (0.76‐1.04)	0.66 (0.46‐0.96)*
pSTAT3^727^ cytoplasm	98 (47)	0.76 (0.46‐1.26)	0.98 (0.53‐1.79)
pSTAT3^705^ nucleus	94 (45)	0.97 (0.85‐1.10)	1.36 (1.05‐1.78)
Pathological Gleason Score			
Low—ISUP 1 and 2	56 (22)	Reference	Reference
High—ISUP 3, 4, and 5	43 (26)	2.99 (1.12‐3.58)*	1.36 (0.72‐2.59)
Pathological T stage			
Low ≤pT2	51 (15)	Reference	Reference
High ≥pT3	48 (33)	3.84 (2.04‐7.23)***	3.08 (1.40‐6.75)**

Abbreviations: BCR, biochemical recurrence; TMA, tissue microarrays.

*N* = number of patients included in analysis; events=BCR.

* *P* < 0.05.

** *P* < 0.01.

*** *P* < 0.001.

^§^ Multivariable analysis with biomarker (tSTAT3 nuc/cyt, pSTAT3^727^ nuc/cyt, and pSTAT3^705^ nuc) scores as continuous variables and pGS and pT stage as low/high.

## 4 DISCUSSION

In the present study we examined tSTAT3, pSTAT3^727^, and pSTAT3^705^ expression in localized hormone naïve PCa to evaluate whether their expression in early stage cancer can be prognostic of disease progression. Surprisingly, we found that both tSTAT3 as well as its phosphorylated forms showed lower expression in the cancer cores, compared with the benign cores from the same patients and that the expression was lowest in higher GS cores in both analyzed cohorts (Malmö and Dublin).

Moreover, our data indicated that the patients with the lower nuclear and cytoplasmic pSTAT3^727^ and nuclear pSTAT3^705^ expression in the cancer cores had shorter time to BCR and therefore worse prognosis. Our data is in line with findings that total tSTAT3 protein expression decreases with increasing Gleason scores^13^ and that pSTAT3^727^ expression is lower in patients with higher pT stages.^19^ However, all of the above is in contrast to previous reports where an increase of pSTAT3^727^ and pSTAT3^705^ in cancerous tissue was observed^20^, ^21^, ^22^, ^23^, ^24^ and high that pSTAT3 levels were indicative of disease progression^25^—see below.

Possible explanations for the discrepancies may lie in the methodology. Our primary cohort was substantially larger (*n* = 300) than those in Dhir et al^21^ (*n* = 42), Mora et al^20^ (*n* = 45), and Campbell et al^22^ (*n* = 21), and we have confirmed our results in an independent cohort of 99 patients (Dublin cohort). It is also important to note that all patients included in our study, in both cohorts, had localized disease and were hormone‐naïve, whereas this information is not available for the other studies. Horinaga et al^25^ did use a similar cohort to ours (*n* = 92) but scored pSTAT3 expression in the tissue overall (as did Dhir et al), without focusing on the epithelial cells or specifically cancer regions. In our study, we focused on the STAT3 expression in the glandular epithelium, and distinguished between cancer and benign areas. Considering the involvement of pSTAT3 in inflammation, it is possible that the presence of pSTAT3 in the inflammatory infiltrate and the microenvironment can dictate the progression of the disease.^26^ pSTAT3 expressing infiltrating immune cells have been observed in tumors of high risk PCa patients.^27^ It would be interesting to study pSTAT3 expression specifically in the stroma and the inflammatory infiltrate in PCa tissue. It would also be crucial to investigate samples from patients with more advanced disease and therapy resistant PCa, as STAT3 is implicated in drug resistance^28^, ^29^ to see whether their pSTAT3 expression is higher. Our “benign” cores come from benign areas of cancerous prostates. It is possible that a “field effect” occurs, and the distant benign areas are affected by the cancer in the whole prostate. Investigating the pSTAT3 expression patterns in benign prostatic tissue with a confirmed nonmalignant follow‐up would be of interest. Lower pSTAT3 levels in biopsies of individuals who were confirmed to be cancer‐free on repeat biopsies, compared with biopsies from patients who developed PCa were reported by Han et al.^30^

Our own recent findings have found high levels of pSTAT3^705^ in bone, lymph node, and other organ metastases,^14^ thus further highlighting the role of STAT3 activation in metastatic disease. An interesting study by Tam et al^12^ compared hormone‐sensitive and hormone‐refractory tumors from the same patients. They proposed that high pSTAT3^705^ in the cytoplasm of hormone‐refractory tumors is prognostic of worse outcome, but not in the nucleus. They also found no correlation of pSTAT3^705^ and pSTAT3^727^ expression (nuclear or cytoplasmic) with Gleason score. What they did find, was that patients with an increase of cytoplasmic pSTAT3^705^ during the progression to hormone‐refractory PCa had worse prognosis than those who had no changes or decrease in the cytoplasmic pSTAT3^705^, although examples of such staining are lacking in their publication. In the present study, we did not observe any cytoplasmic pSTAT3^705^ staining in neither of our two cohorts. It seems plausible that cytoplasmic pSTAT3 staining is detectable at later stages of the disease and that pSTAT3 may have different effects in more advanced PCa. We did, however, find cytoplasmic expression of both pSTAT3^727^ and tSTAT3. Low cytoplasmic pSTAT3^727^ was associated with a shorter time to BCR. Conversely, higher cytoplasmic tSTAT3 was associated with shorter time to BCR in the Dublin cohort.

There is evidence that unphosphorylated STAT3 (uSTAT3) can enhance transcription and may be involved in oncogenesis.^31^ This may explain why in our study, tSTAT3 does not follow the same patterns in relation with BCR as the two pSTAT3 variants. Interestingly, while pSTAT3^727^ and pSTAT3^705^ were highly correlated with each other, the correlation with tSTAT3 in cancer cores was a lot lower. This indicates that some other factors, apart from phosphorylated STAT3 contribute to tSTAT3. Some involvement of uSTAT3 may be possible, but unfortunately we have no way to detect it with immunohistochemistry (IHC).

Moreover, the protein expression levels of pSTAT3 measured by IHC, may not reflect the exact transcriptional activity of STAT3, as STAT3 has two splice variants: α and β.^10^ STAT3β is suggested to function as a tumor suppressor and a negative regulator of STAT3α which has mainly tumor promoting activities.^32^ In the current study we cannot differentiate STAT3α from STAT3β with the antibodies used. In a study of esophageal squamous cell carcinoma, high pSTAT3α was correlated to longer overall survival, but in combination with low pSTAT3β, the outcome was worse.^33^

It is also important to consider that coexpression of STAT3 with other intracellular mediators may be of clinical interest.^34^ Pencik et al^13^ showed that low tSTAT3 was correlated with a poor outcome, which was worse if combined with low p14^ARF^ expression. Similarly, studies in glioblastoma found that pSTAT3 can have pro‐oncogenic or tumor‐suppressive functions depending the presence of PTEN.^35^ Therefore more insight into the different STAT3 splice variants and the involvement of other STAT3 cofactors such as ARF, PTEN or SOCS3 may be needed in future studies.

The two cohorts used from the two different sites were similar (Table 1), with the Dublin cohort encompassing some more advanced stage patients (pGS ≥ 8, 26%) and more BCR events (49%) compared with the Malmö cohort (pGS ≥ 8, 15%; BCR 28%). The Dublin cohort was three times smaller than the Malmö cohort and had a shorter follow‐up time (average 52 months, compared with 130 in the Malmö cohort). This may explain why the Dublin cohort, although showing similar trends to Malmö, did not reach significance in most cases.

As a control for the cohort, we examined the prognostic values of pathologic GS and pT stage. In the Malmö cohort they both correlated with survival, showing that those two are the best prognostic markers for BCR. In the Dublin cohort, while the pT stage showed expected BCR patterns, the pGS was not prognostic of BCR. This discrepancy is likely to be the result of the patients being selected and matched in the Dublin cohort, resulting in a 49% rate of BCR, which is unnaturally high for a normal, unselected population. This may explain some of the differences between the cohorts although it is impossible to tell how much these factors influence the results. It is very difficult to obtain similar material from different centers.

pGS and pT stage, together with factoring in the patients age, provide good models for BCR prediction. Adding our results of epithelial tSTAT3 and pSTAT3 expression lowered the prognostic value of pGS and pT stage, and therefore pSTAT3 expression is unlikely to be beneficial as a prognostic marker in hormone naïve localized PCa (Table 3).

## 5 CONCLUSIONS

Low pSTAT3^705^ and pSTAT3^727^ expression in epithelial cells of cancerous prostatic glands in hormone‐naïve PCa was found to be associated with shorter time to BCR. However, pSTAT3^705^, pSTAT3^727^, and tSTAT3 expression did not improve the prognostic value of pGS and pT stage and overall, may not be good prognostic biomarkers in early stage PCa.

## CONFLICT OF INTERESTS

AB and RH are cofounders and shareholders in Glactone Pharma AB.

## Supporting information

Supporting information

Supporting information

Supporting information

Supporting information
