# Supplementary figures and images for: Expression of tSTAT3, pSTAT3727, and pSTAT3 705 in the epithelial cells of hormone‐naïve prostate cancer
Source: Prostate. 2019 Mar 24;79(7):784–97. doi: 10.1002/pros.23787 (PMC6766958; doi:10.1002/pros.23787)

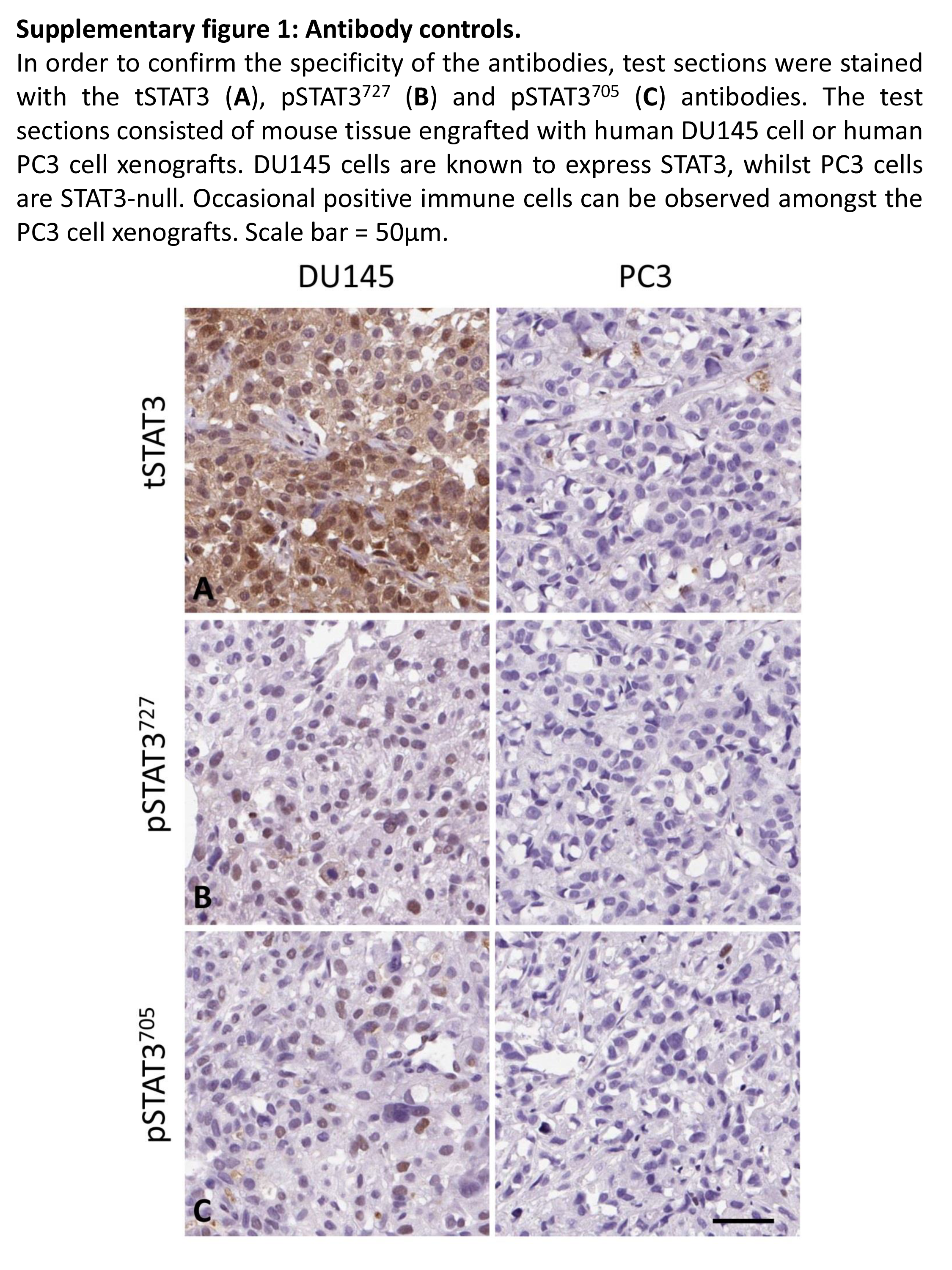

Supplement: Supplementary file 1 — Supporting information [file PROS-79-784-s001.tif]

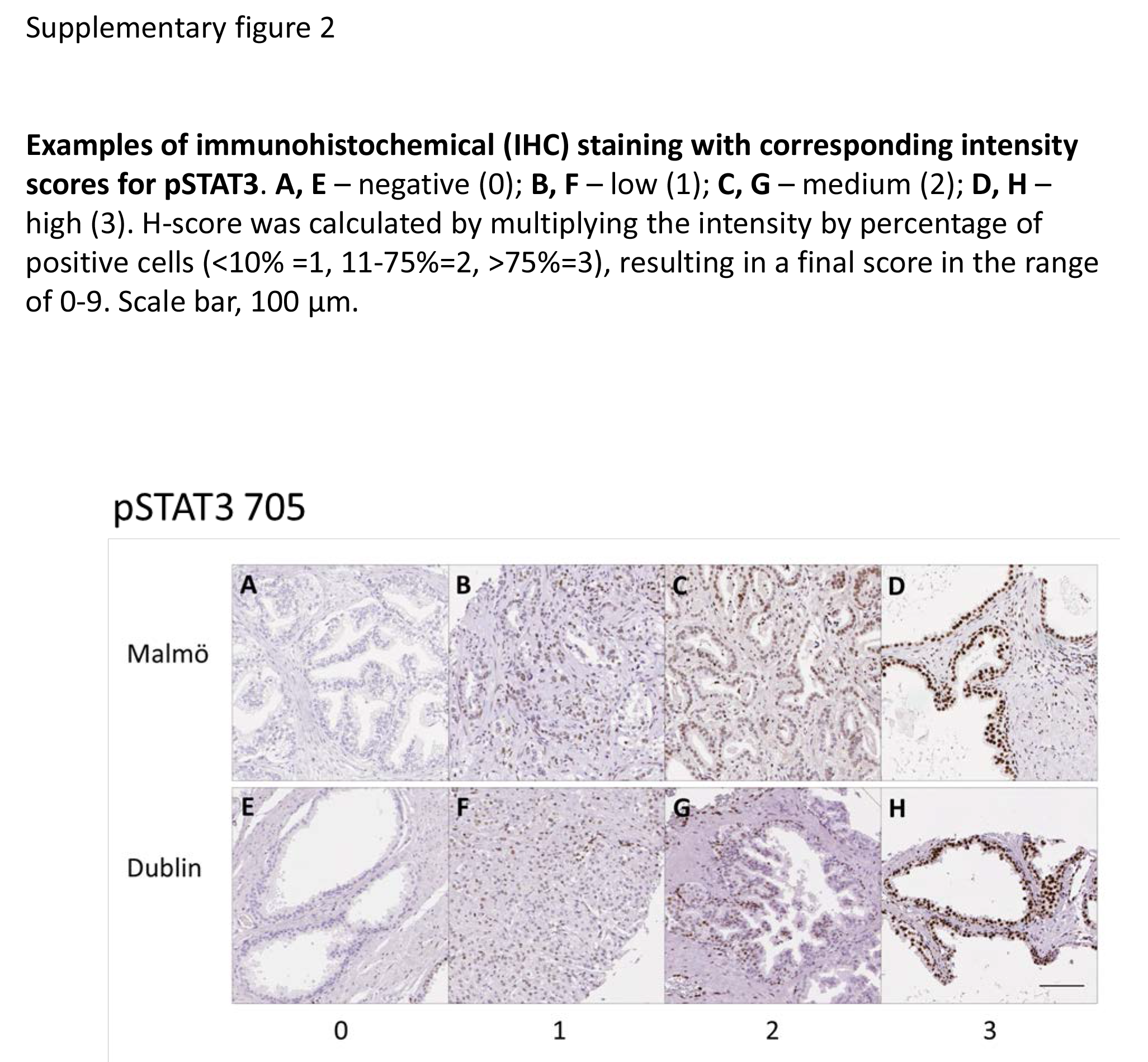

Supplement: Supplementary file 2 — Supporting information [file PROS-79-784-s002.tif]

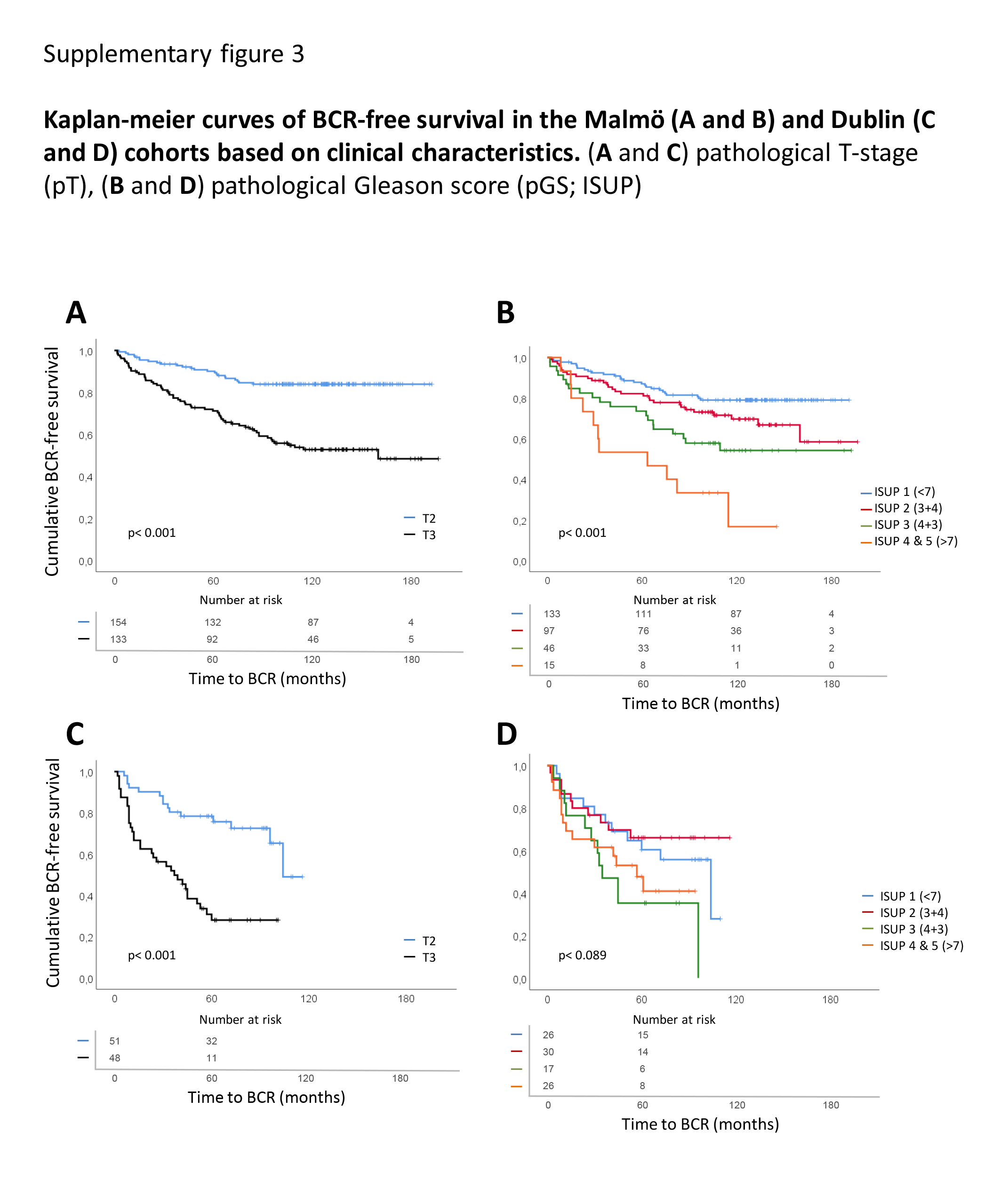

Supplement: Supplementary file 3 — Supporting information [file PROS-79-784-s003.tif]
