# Supplementary material for: Expression of tSTAT3, pSTAT3727, and pSTAT3 705 in the epithelial cells of hormone‐naïve prostate cancer
Source: Prostate. 2019 Mar 24;79(7):784–97. doi: 10.1002/pros.23787 (PMC6766958; doi:10.1002/pros.23787)
Supplement: Supplementary file 4 — Supporting information [file PROS-79-784-s004.docx]

Supplementary figures

**Supplementary Figure 1.**

**Antibody controls.** In order to confirm the specificity of the antibodies, test sections were stained with the tSTAT3 (**A**), pSTAT3^727^ (**B**) and pSTAT3^705^ (**C**) antibodies. The test sections consisted of mouse tissue engrafted with human DU145 cell or human PC3 cell xenografts. DU145 cells are known to express STAT3, whilst PC3 cells are STAT3-null. Occasional positive immune cells can be observed amongst the PC3 cell xenografts. Scale bar = 50µm.

**Supplementary Figure 2.**

**Examples of immunohistochemical (IHC) staining with corresponding intensity scores for pSTAT3**. **A, E** – negative (0); **B, F** – low (1); **C, G** – medium (2); **D, H** – high (3). H-score was calculated by multiplying the intensity by percentage of positive cells (<10% =1, 11-75%=2, >75%=3), resulting in a final score in the range of 0-9. Scale bar, 100 µm.

**Supplementary Figure 2.**

**Kaplan-meier curves of BCR-free survival in the Malmö (A and B) and Dublin (C and D) cohorts based on clinical characteristics.** (A and C) pathological T-stage (pT), (B and D) pathological Gleason Score (pGS) stratified by the ISUP 2014 Gleason grade classification.
